# A frame of wideband wireless signal recognition and parameter extraction based on semantic segmentation

**DOI:** 10.1371/journal.pone.0346685

**Published:** 2026-04-27

**Authors:** Lulu Liu, Rui Zhu, Peng Chu, Zhibo Shi, Juan Tian, Yushuai Zhang, Le Gao, Yaru Li

**Affiliations:** 1 Xi’an Key Laboratory of High-Precision Industrial Intelligent Vision Measurement Technology, Xi’an, China; 2 School of Electronic Information, Xijing University, Xi’an, China; Beijing Institute of Technology, CHINA

## Abstract

With the rapid development of wireless communication technologies, spectrum resources are becoming increasingly scarce, and spectrum monitoring technologies targeting control and interference suppression impose higher requirements on the real-time performance, reliability, and intelligence of signal detection, recognition, and key parameter extraction. Traditional signal processing methods heavily rely on operators’ prior knowledge, making it difficult to achieve intelligent spectrum monitoring, and often exhibit poor performance in complex electromagnetic environments with unknown signals or strong interference. Existing deep learning-based automatic modulation recognition techniques are more focused on signal recognition, with relatively limited research on detection and key parameter extraction. To address these challenges, this paper proposes a wideband signal processing frame based on semantic segmentation and signal spectrogram. The frame employs RepViT as the backbone network and achieves detection, recognition, and key parameter extraction of wideband signals through precise semantic segmentation of signal spectrogram. Experimental results on a large-scale synthetic dataset demonstrate that the proposed frame achieves a maximum signal recognition rate (mAcc) of 82.43% and an average signal recognition rate (aAcc) of 65.16% in multi-modulation scenarios and under different noise power levels. In terms of parameter extraction, the normalized root mean squared error (NRMSE) for time parameters (e.g., start time, and duration) is controlled within the ranges of 0.3%−2.8% and 0.4%−1.6%, respectively, while the NRMSE for frequency parameters (e.g., center frequency, and bandwidth) reaches 8.7% and 0.6% in multi-classification tasks, providing an effective reference solution for intelligent wireless signal analysis.

## 1. Introduction

Blind signal recognition technology is of paramount importance in modern wireless communications, particularly for monitoring and managing signals in non-cooperative or crowded spectrum environments. With the rapid development of fifth-generation (5G) and sixth-generation (6G) mobile communication technologies, spectrum resources are becoming increasingly scarce, and signal environments are growing more complex. These developments impose higher demands on spectrum monitoring, signal recognition, and electromagnetic spectrum management. In scenarios such as dynamic spectrum access and interference management, accurately identifying unknown signal types and parameters is a core task for efficient spectrum utilization and electromagnetic environment awareness, directly related to the performance and reliability of communication systems. Therefore, developing efficient and accurate blind signal recognition technology has become a critical requirement in the fields of communication technology and intelligent spectrum management.

Traditional blind signal recognition techniques mainly rely on signal processing theory, such as Fourier Transform (FT), Wavelet Transform (WT), and Short-Time Fourier Transform (STFT) for time-frequency analysis. These methods are combined with manually designed feature extraction and pattern recognition algorithms. These methods perform well in simple environments but exhibit limitations in practical applications. First, recognition rates are low, particularly in complex electromagnetic environments with low signal-to-noise ratios, multiple coexisting signals, and dynamic interference, where traditional methods often fail to meet practical application requirements. Second, high human involvement is required, necessitating experienced operators to manually set thresholds and rules based on signal characteristics, lacking adaptive capabilities. Finally, strong dependence on skilled personnel, as traditional methods struggle to handle diverse signal characteristics and cannot achieve 24-hour continuous monitoring, limiting their application in automated monitoring systems.

The rapid development of deep learning technology has provided new solutions for blind signal recognition. Deep learning-based signal recognition methods can achieve 24-hour continuous monitoring, which substantially improves the automation and intelligence levels of signal recognition systems. In recent years, modulation recognition methods based on Convolutional Neural Networks (CNN) and Recurrent Neural Networks (RNN) have achieved promising results on datasets such as RadioML, demonstrating the tremendous potential of deep learning in signal recognition tasks.

However, existing deep learning-based blind recognition methods still have certain limitations. Current research primarily focuses on modulation recognition tasks for narrowband signals, assuming that a data segment contains only a single signal and requiring only the identification of that signal’s modulation scheme. However, in practical wideband spectrum monitoring scenarios, wideband received signals often simultaneously contain multiple signals of different modulation types, which may overlap or be adjacent in both time and frequency domains. Existing methods cannot simultaneously accomplish detection, recognition, and parameter extraction for multiple signals, limiting their practical utility.

To address the aforementioned issues, this paper proposes a wideband wireless signal processing frame based on semantic segmentation and signal spectrogram. The frame adopts semantic segmentation methods, transforming the segmentation problem of signal spectrogram into a pixel-level classification problem, enabling simultaneous detection, localization, and recognition of multiple signals. By combining lightweight neural network architectures with Feature Pyramid Networks (FPN), precise segmentation of multiple signals in wideband signals is achieved. Furthermore, this frame proposes an automatic extraction method from segmentation results to signal parameters, achieving automatic estimation of key signal parameters including start time, duration, center frequency, and bandwidth.

The main contributions of this paper include:

1. **RepViT for wideband signal spectrogram segmentation**: We employ a RepViT backbone as the core of a semantic segmentation frame for wideband wireless signal spectrograms. This enables pixel-level detection, localization and modulation-type recognition of multiple coexisting signals in a single wideband snapshot, which goes beyond conventional narrowband single-signal modulation recognition.
2. **Segmentation-driven parameter extraction pipeline**: On top of the RepViT–FPN segmentation head, we design a generic decoding pipeline that converts segmentation masks into structured signal parameters (start time, duration, centre frequency and bandwidth) via connected-component analysis and time–frequency mapping, thus bridging semantic segmentation outputs with traditional signal-parameter estimation.
3. **Systematic comparison with representative lightweight baselines**: Within a unified segmentation and decoding frame, we perform a comprehensive comparison between RepViT and several representative lightweight backbones, including RepUNet, ResNet18 and PoolFormer-S12, on a large synthetic dataset with nine modulation types and eight noise power levels. This provides a first benchmark of lightweight semantic-segmentation-based approaches for wideband signal detection, recognition and parameter extraction.

This paper is organized as follows. Section 2 systematically reviews related research on semantic segmentation techniques, lightweight neural network architectures, signal spectrogram analysis methods, and signal parameter extraction techniques, analyzing the limitations of existing research and proposing corresponding solutions. Section 3 introduces the overall methodology and technical details, dataset construction methods, and the RepViT model architecture. Section 4 presents experimental results for binary and multi-classification tasks, comparing and analyzing the performance of different models, and evaluating the accuracy of signal parameter extraction. Finally, Section 5 summarizes the main findings and contributions of this research, proposing future research directions and improvement suggestions.

## 2. Related work

Traditional blind signal recognition methods are mainly based on signal processing theory and statistical pattern recognition techniques. In time-frequency analysis, STFT achieves local Fourier transforms through sliding windows to obtain time-frequency spectra [1]. Continuous Wavelet Transform (CWT) utilizes multi-scale mother wavelets to enhance time-frequency resolution [2], while Wigner-Ville Distribution (WVD) provides high-precision instantaneous frequency estimation in bilinear form but is prone to introducing cross-term interference [3]. At the feature extraction level, researchers often design higher-order cumulant features to enhance resistance to Gaussian noise [4], and combine cyclostationary statistics to capture the periodic structure of modulated signals [5]. In the classification stage, Support Vector Machines (SVM) achieve robust decisions through the maximum margin principle [6], while Random Forest (RF) and K-Nearest Neighbors (KNN) algorithms accomplish multi-class discrimination Identification through ensemble learning and distance metrics, respectively [7,8], and Hidden Markov Models (HMM) are used to model temporal dependencies of sequential signals [9]. Although the aforementioned methods perform well in ideal scenarios with high signal-to-noise ratios and single signals, their dependence on manual features and empirical thresholds causes them to face performance bottlenecks in complex environments with low signal-to-noise ratios and multiple coexisting signals.

With the rapid development of deep learning technology, deep learning-based signal modulation recognition methods have gradually become a research hotspot. O’Shea, T. J. et al. [10] first applied CNN to modulation recognition tasks, achieving performance superior to traditional methods on the RadioML dataset. This method directly uses I/Q sampled data or spectrogram of signals as input, automatically learning signal feature representations through convolutional layers, avoiding the limitations of manual feature design.

Lin et al. [11] conducted a systematic survey of deep learning techniques in automatic modulation recognition. They summarized network architectures and training strategies that maintain high recognition rates under different signal-to-noise ratio conditions, further demonstrating the advantages of deep learning methods in complex electromagnetic environments. RNN and Long Short-Term Memory (LSTM) networks excel in sequential signal analysis. RNN can utilize the temporal characteristics of signals, capturing temporal dependencies through recurrent connections. LSTM [12] address the gradient vanishing problem of RNN through gating mechanisms, enabling the processing of long sequences. Bidirectional Long Short-Term Memory (BiLSTM) networks further improve recognition performance by simultaneously considering forward and backward information. In recent years, Transformer architectures have also been applied to signal recognition tasks, with Vision Transformer (ViT) achieving efficient feature representation through self-attention mechanisms for global modeling of split patches [13].

However, the high computational complexity of Transformers often hinders their deployment in resource-constrained scenarios. To improve computational efficiency while maintaining accuracy, lightweight neural network architectures have been widely applied to signal recognition tasks. The MobileNet series [14,15] reduces parameters and computational costs through depthwise separable convolutions, achieving real-time inference on mobile devices. EfficientNet [16] achieves a balance between accuracy and efficiency through compound scaling strategies. MobileViT [17] combines ViT’s global modeling capability with CNN’s inductive bias, reducing computational complexity while maintaining performance.

Despite progress in deep learning-based modulation recognition methods, existing research primarily focuses on narrowband signal modulation recognition, assuming single-signal scenarios. However, practical wideband spectrum monitoring requires simultaneous detection, localization, and recognition of multiple signals, which existing methods cannot accomplish.

Semantic segmentation technology, as an important branch of computer vision, aims to assign semantic category labels to each pixel in an image. Applying semantic segmentation technology to signal spectrogram analysis enables pixel-level precise classification, simultaneously accomplishing detection, localization, and recognition of multiple signals, providing a new technical path for the automated analysis of wideband signals.

The proposal of Fully Convolutional Networks (FCN) marked the beginning of deep learning in semantic segmentation. Long et al. [18] first replaced fully connected layers with convolutional layers, achieving end-to-end semantic segmentation. U-Net [19] achieved multi-scale feature fusion through encoder-decoder architecture and skip connections, achieving remarkable success in medical image segmentation. The DeepLab series [20,21] effectively expanded the receptive field and improved segmentation accuracy by introducing atrous convolution and Atrous Spatial Pyramid Pooling (ASPP) modules. PSPNet [22] further improved segmentation performance by aggregating contextual information at different scales through pyramid pooling modules.

With the rise of ViT, Transformer-based semantic segmentation methods such as SegFormer [23] and Segmenter [24] have demonstrated powerful performance. However, the computational complexity of these methods is high, making them difficult to apply in resource-constrained scenarios. Addressing the lightweight requirements in wireless communications and mobile environments, Zhang et al. [25] surveyed the deployment challenges of deep learning in mobile and wireless networks, highlighting the importance of model compression and hardware-friendly design. The application of semantic segmentation to spectrum monitoring represents a novel approach that combines pixel-level classification with signal processing theory. The Mobile-Former architecture [26] further demonstrates the latest progress in complementary fusion of CNN and Transformers to achieve lightweight design, providing design inspiration for the RepViT-FPN structure adopted in this paper. RepViT [27] is a hybrid model that introduces ViT’s efficient architecture design into CNN. RepViT employs re-parameterization technology, using multi-branch structures during training to enhance expressive capability and fusing into a single branch during inference to improve speed. Combined with depthwise separable convolutions and the Mobile-Former hybrid architecture, RepViT achieved over 80% top-1 accuracy on ImageNet classification tasks while achieving 1ms inference latency (on iPhone 12).

The RepVGG work [28] systematically analyzed the theoretical foundations and performance benefits of re-parameterized convolutions, demonstrating the feasibility of equivalently transforming complex training structures into single-branch networks during inference, providing theoretical support for the fast inference of the method in this paper.

EfficientFormer [29] combines Transformer’s global modeling capability with CNN efficiency by designing efficient Token Mixers. PoolFormer [30] uses simple pooling operations to replace self-attention mechanisms, reducing computational complexity while maintaining performance. ResNet [31] addresses the gradient vanishing problem in deep networks through residual connections, with ResNet18 as a lightweight version maintaining good performance while having a smaller number of parameters. UNet variants are widely used in semantic segmentation tasks, achieving multi-scale feature fusion through encoder-decoder architecture and skip connections, with lightweight UNet variants reducing model complexity through techniques such as depthwise separable convolutions and channel compression.

The application of semantic segmentation technology to signal spectrogram analysis is relatively new and still requires further exploration in data scale, model design, and application scenarios. In signal parameter extraction, traditional methods typically extract parameters based on time-frequency analysis results through threshold segmentation, peak detection, and edge detection methods. Deep learning-based parameter extraction methods typically adopt end-to-end learning approaches, directly predicting parameters from raw signals or spectrogram, but require large amounts of labeled data and are difficult to interpret regarding the model’s decision process. Parameter extraction methods based on segmentation results combine the segmentation capability of deep learning with traditional signal processing theory, extracting signal parameters from segmentation masks through connected component analysis, frequency mapping, and geometric feature extraction, offering good interpretability and robustness.

A systematic review of the literature reveals that research on applying semantic segmentation to signal spectrograms remains limited, particularly concerning lightweight model architectures. To address this gap, this paper selects representative models including RepViT, RepUNet, ResNet18, and PoolFormer-S12 to conduct research on wideband signal detection, recognition, and parameter extraction based on semantic segmentation.

## 3. Method

### Overview of the proposed frame

**Fig 1:**
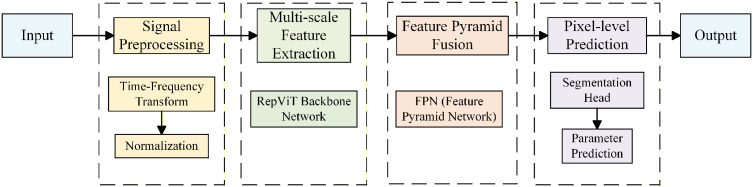
Overall frame architecture.

The overall frame proposed in this paper comprises four main steps: wideband reception, time-frequency transformation, semantic segmentation, and center frequency and bandwidth parameter calculation. First, the wideband reception system collects wideband signal data containing multiple signals. Second, time-domain signals are converted to spectrogram through STFT, achieving joint representation of signals in both time and frequency domains. Then, a RepViT-based semantic segmentation model performs pixel-level classification on spectrogram, achieving detection, localization, and recognition of multiple signals. Finally, key parameters such as center frequency and bandwidth are extracted from the segmentation results for each signal. The overall system architecture is shown in Fig 1.

In the first stage, to simulate real wideband reception scenarios, this study simultaneously activated multiple modulation schemes during signal generation and randomly sampled center frequencies, symbol rates, duration, and start times to form composite wideband signals. The generated baseband signals are upconverted to radio frequency form, and then Additive White Gaussian Noise (AWGN) is superimposed to simulate different noise power levels. The final output time-domain complex signal is denoted as *s*(*t*),providing input for subsequent time-frequency transformation. This stage emphasizes signal diversity and noise controllability, providing training samples covering multiple modulation types and different signal-to-noise ratios for the semantic segmentation model.

In the time-frequency transformation stage, wideband wireless signals need to be converted to spectrogram for pixel-level recognition. This study employs STFT to construct time-frequency representations, with the formula as follows:


$$STFT(t,f)=\int_{-\infty }^{+\infty }s(\tau )\omega (\tau -t)e^{-j2\pi f\tau }d\tau$$ (1)

Where ω(·) is the Hamming window. In our implementation, STFT is performed using the MATLAB’s pspectrum function with a time resolution of 0.01 s. The sampling rate is *f*_*s*_ = 2.4 MHz and the duration of the analysis window is *T* = 1.8 s. The resulting spectrogram has dimensions of *H* = 1024 (frequency bins) and *W* = 741 (time frames), with a frequency resolution of $f_{res}=f_{s}/H\approx 2.34$ kHz per pixel and a time resolution of 0.01 s per time frame. These parameters are chosen to balance frequency resolution (for accurate bandwidth estimation) and time resolution (for precise start/stop time localization). The resulting magnitude spectrum is converted to the power domain and normalized, yielding a single-channel spectrogram matrix. To enhance the visual expression of time-frequency features and adapt to RGB input format, each element in the spectrogram power spectrum is mapped to RGB color space according to amplitude magnitude through a predefined color mapping function, with low amplitude values corresponding to dark colors (e.g., blue) and high amplitude values corresponding to bright colors (e.g., red), forming spectrogram with rich color gradients. Finally, a three-channel spectrogram $I\in {\mathbb{R}}^{H\times W\times 3}$ of size *H* × *W* is obtained.

In the semantic segmentation stage, the model aims to classify each pixel of the three-channel spectrogram, outputting a segmentation mask $M\in {\mathbb{R}}^{H\times W\times C}$. For pixel(*i*,*j*), the probability of category given by the semantic segmentation model $f_{\theta }$ is:


$$P(c|I,i,j)=f_{\theta }(I{)}_{i,j,c}$$ (2)

Where *c* is the number of categories (including background and various modulation types).

In the center frequency and bandwidth parameter calculation stage, the masks output by semantic segmentation need to be further converted into structured parameters. First, connected component analysis is performed, converting multi-class masks to binary masks and extracting signal region sets $R=\{R_{1},R_{2},\cdots ,R_{N}\}$, filtering noise clusters with areas smaller than threshold K pixels. Subsequently, frequency mapping and parameter estimation are completed. The pixel coordinate in the frequency direction is mapped to the physical frequency:


$$f=f_{min}+\frac{H-y-1}{H}\times (f_{\max}-f_{\min})$$ (3)

Where *f*_*min*_ and *f*_*max*_ are the minimum and maximum values of the frequency axis, respectively, and *H* is the height of the time-frequency spectrogram in the frequency direction. The center frequency is obtained through the frequency centroid of pixels in each connected component, while the bandwidth is calculated from the difference between the maximum and minimum values in the frequency direction. To establish a one-to-one correspondence between predicted signals and ground truth signals, a matching strategy based on time and frequency distance is adopted:


$$d(p_{i},g_{i})=\alpha |t_{p_{i}}-t_{g_{i}}|+\beta |f_{p_{i}}-f_{g_{i}}|$$ (4)

Where α and β are weight coefficients for time and frequency, respectively. In practice, we set α=0.6 and β=0.4, giving slightly higher weight to time-domain alignment, since start and end times are typically more sharply defined than frequency boundaries in the spectrogram. The time and frequency tolerances are set to 0.1 s and 50 kHz, respectively. A simple sensitivity analysis, where α is varied from 0.4 to 0.8 (with β=1−α), shows that the overall NRMSE of all four parameters changes by less than 2%, indicating that the matching performance is not overly sensitive to the exact choice of α and β. When the number of predictions matches the number of ground truth, a direct matching scheme sorted by start time is preferentially adopted.

### Dataset

All experiments in this study are conducted on a synthetic dataset generated from analytical signal models with additive white Gaussian noise (AWGN). This design allows for controlled evaluation under various noise levels and signal configurations. However, real-world RF signals may contain additional impairments (e.g., multipath fading, non-Gaussian noise, hardware distortions), which are not explicitly modeled here. This study constructed a signal dataset containing 9 modulation types, including 6 digital modulations (BPSK, QPSK, 8PSK, 16QAM, 64QAM, MSK) and 3 analog modulations (FM, AM-DSB, AM-SSB).

**Fig 2:**
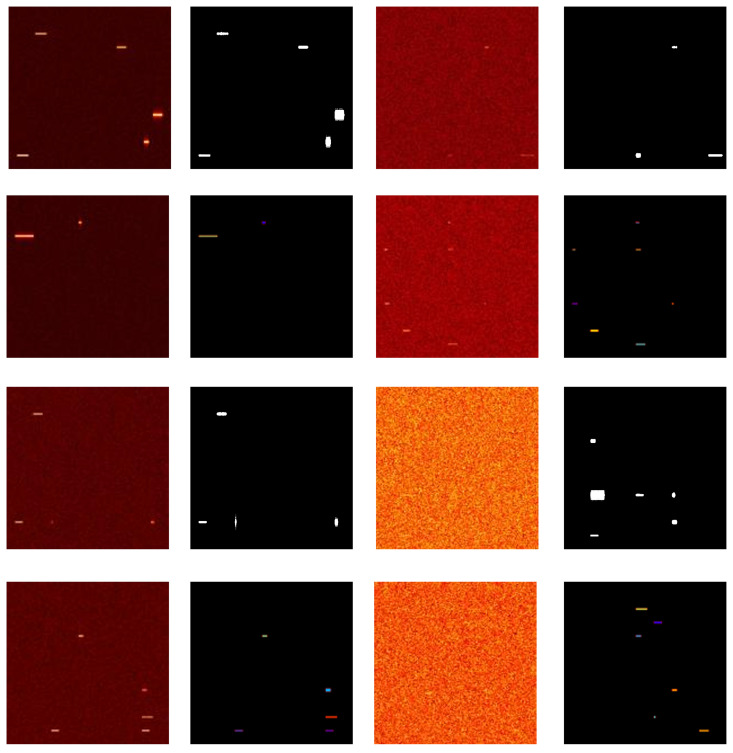
Dataset examples: (a) Binary classification (spectrogram and labels), (b) Multi-classification (spectrogram and labels).

Fig 2 shows typical spectrogram examples in the dataset, including spectrogram and their corresponding labels under different modulation types and noise levels.For binary classification (a), the left side shows spectrograms and the right side shows corresponding binary labels, with white regions representing signals and black regions representing background. For multi-classification (b), the left side shows spectrograms and the right side shows corresponding multi-class labels, with different colors representing different modulation types and black regions representing background.

The figure shows spectrogram under noise levels of 0dBW, 10dBW, 20dBW, and 30dBW from top to bottom, showing that signal features gradually blur as noise intensity increases. It also displays typical spectrogram features of 9 randomly generated modulation types. Unlike binary classification labels where black represents background and white represents modulated signals, it should be noted that multi-classification label files themselves use integer index encoding: 0 represents background, and 1–9 correspond to nine modulation types, respectively. If viewed using grayscale or fixed threshold methods, they will visually appear as almost entirely black low-contrast images, easily mistaken for missing category information. To facilitate verification of label generation, different colors are used here to represent labels of different modulation schemes.

During signal generation, center frequencies are randomly selected from a preset center frequency set *F*_*c*_, symbol rates are randomly selected from a preset symbol rate set *R*_*s*_, symbol durations are randomly selected from a preset symbol duration set *T*_*d*_ and start times are randomly selected from a preset start time set *T*_*s*_. This study employs a sampling rate *f*_*s*_, mapping center frequencies to the baseband range $\left[-\frac{f_{s}}{2},\frac{f_{s}}{2}\right]$, through modulo operation, with the mapping formula:


$$f_{c,eq}=f_{c}modf_{s}$$ (5)

The mathematical expression for QAM-type modulations (including BPSK, QPSK, 8PSK, 16QAM, 64QAM) signals is:


$$s_{QAM}(t)=\Sigma_{n}b_{n}p(t-nT_{s})e^{j(2\pi f_{c}t+\phi_{n})}$$ (6)

Where *b*_*n*_ is the baseband signal of the modulated signal, and $\phi_{n}$ is the initial phase. *p*(*t*) is the raised cosine shaping pulse.

The mathematical expression for MSK signals is:


$$s_{MSK}(t)=cos(2\pi f_{c}t+\phi t)$$ (7)

Where *f*_*c*_ is the carrier frequency, and the phase ϕ(t) is:


$$\phi (t)=\phi (0)+\pi \Sigma_{n}a_{n}p(t-nT_{s})$$ (8)

Where *a*_*n*_ is the value of the *n*-th digital symbol. The generation method for analog modulation signals is as follows. The mathematical expression for FM signals is:


$$s_{FM}(t)=A_{c}cos\left(2\pi f_{c}t+2\pi f_{dev}\int_{0}^{t}m\tau d\tau \right)$$ (9)

Where *f*_*dev*_ is the frequency deviation coefficient, m(τ) is the original baseband message signal of the modulated signal (a function varying with time τ), *A*_*c*_ is the carrier amplitude. The mathematical expression for AM-DSB signals is:


$$s_{AM-DSB}(t)=A_{c}[1+m(t)]\cos\left(2\pi f_{c}t\right)$$ (10)

Where *m*(*t*) is the original baseband message signal of the modulated signal. AM-SSB signals suppress one sideband of AM-DSB through Hilbert transform, with the mathematical expression:


$$s_{AM-SSB}(t)=A_{c}[m(t)\cos(2\pi f_{c}t)\mp \hat{m}(t)sin(2\pi f_{c}t)]$$ (11)

Where $\hat{m}(t)$ is the Hilbert transform of *m*(*t*). Furthermore, the received signal which is also modulated *t*hrough Eq (10)-(11) is:


r(t)=s(t)+n(t) (12)

Where *s*(*t*) is the modulated signal and:


$$n(t)=n_{I}(t)+jn_{Q}(t)$$ (13)

*n*(*t*) is complex Gaussian white noise, with *n*_*I*_(*t*), $n_{Q}(t)\sim N(0,\sigma_{n}^{2})$, and the noise power of *n*(*t*) is $P_{n}=2\sigma_{n}^{2}$. The noise power levels are specified in decibels relative to 1 Watt (dBW). Let *P*_*n*_ denote the average noise power of *t*he complex Gaussian noise *n*(*t*); then $P_{n}(\text{dBW})=10\log_{10}(P_{n}/1\text{ W})$. In our experiments, the average signal power *P*_*s*_ is kept constant, while the noise power *P*_*n*_ is varied to obtain eight noise conditions: −5, 0, 5, 10, 15, 20, 25 and 30 dBW. The corresponding signal-to-noise ratio (SNR) can be expressed as SNR(dB) = *P*_*s*_(dBW) − *P*_*n*_(dBW). For example, if the signal power *P*_*s*_ = 0 dBW, then a*t* noise power *P*_*n*_ = −5 dBW, the SNR is 0 − (−5) = 5 dB, while at *P*_*n*_ = 30 dBW, the SNR is 0 − 30 = −30 dB. The dataset is generated under these 8 different noise power levels. The dataset is generated under 8 different noise power levels. Spectrograms are generated through Eq (1), with spectrogram matrix dimensions of *H* × *W* pixels, frequency resolution *f*_*res*_ = *f*_*s*_/*H* pixels, and time resolution determined according to STFT window length and total signal duration.

The dataset is divided into binary classification and multi-classification. Binary classification labels only distinguish signals from noise, while multi-classification distinguishes specific modulation schemes. The binary classification dataset contains approximately 500 samples per noise level, and the multi-classification dataset contains approximately 14,400 samples per noise level, with a total of over 100,000 spectrogram samples, divided into training, validation, and test sets in an 8:1:1 ratio.

In the signal generation process, multiple signals can coexist within a single spectrogram, but they are constrained to be non-overlapping in both time and frequency domains. This design ensures that each signal occupies distinct time–frequency regions, facilitating clear signal separation and parameter extraction. The number of signals per spectrogram varies, with each sample containing one or more signals of different modulation types, all maintaining non-overlapping time–frequency boundaries.

### Model architecture

This paper adopts RepViT as the backbone for semantic-segmentation-driven signal parameter extraction. RepViT is a hybrid CNN/ViT architecture whose structural re-parameterization supplies rich multi-branch representations during training while collapsing to an efficient single-branch form during inference. Built on this backbone, the proposed signal recognition frame establishes a coherent “semantic segmentation → parameter recovery” loop: the STFT-based spectrogram $I\in {\mathbb{R}}^{H\times W\times 3}$, is encoded into multi-scale descriptors, an FPN head composes a dense mask M, and connected-component analysis with time–frequency mapping yields start time, duration, center frequency, and bandwidth for each detected emission.

#### Backbone feature hierarchy.

**Fig 3:**
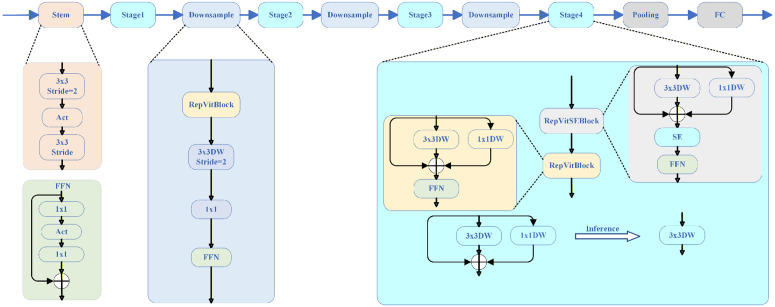
RepViT-based architecture.


As illustrated in Fig 3, the backbone consists of a Patch Embedding stem followed by four RepViT stages. The input time-frequency spectrogram $I\in {\mathbb{R}}^{H\times W\times 3}$ is progressively transformed through these stages, with spatial resolution halving and channel width expanding at each stage. The multi-scale feature outputs can be formally expressed as:

$$F=F_{i}|F_{i}\in {\mathbb{R}}^{H/S_{i}\times W/S_{i}\times C_{i}},S_{i}\in \{4,8,16,32,32\}$$ (14)

Where $F_{0}\in {\mathbb{R}}^{H/4\times W/4\times C_{0}}$ is the output of Patch Embedding (stride-2 3 × 3 convolution), $F_{1}\in {\mathbb{R}}^{H/4\times W/4\times C_{1}}$ is from Stage 1 (stride-1 blocks), $F_{2}\in {\mathbb{R}}^{H/8\times W/8\times C_{2}}$ from Stage 2 (first block stride 2), $F_{3}\in {\mathbb{R}}^{H/16\times W/16\times C_{3}}$ from Stage 3, and $F_{4},F_{5}\in {\mathbb{R}}^{H/32\times W/32\times C_{4/5}}$ from Stage 4.

This multi-scale hierarchy preserves both coarse energy envelopes and fine spectral structures, providing the necessary context for precise signal localization and parameter estimation.

#### RepViTBlock structure.

**Fig 4:**
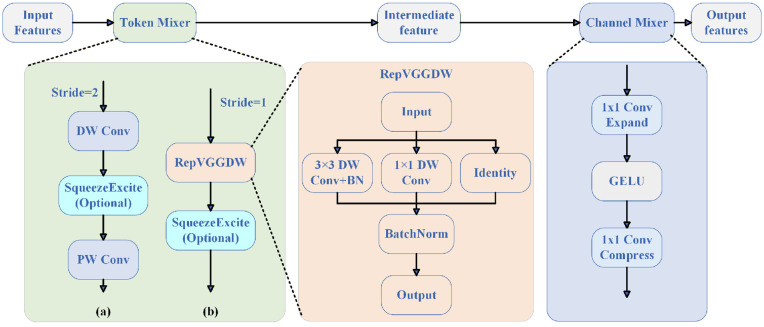
RepViTBlock structure: (a) stride-2 block, (b) stride-1 block.

RepViT blocks (RepViTBlock) are the basic building units of RepViT for processing signal time-frequency features, consisting of two components: Token Mixer and Channel Mixer. Fig 4 shows the detailed structure of RepViTBlock. For stride-2 blocks (a), the Token Mixer adopts a downsampling structure consisting of 3×3 depthwise separable convolution with stride 2, SE attention module, and 1×1 point convolution. For stride-1 blocks (b), the Token Mixer adopts RepVGGDW structure with 3×3 depthwise separable convolution, 1×1 point convolution, and residual connection.

The Token Mixer is responsible for spatial mixing, enabling the model to capture relationships among adjacent time–frequency regions. For stride-1 blocks the RepVGGDW form is used:


$$F_{out}=BN(DWConv_{3\times 3}(F_{in})+Conv_{1\times 1}(F_{in})+F_{in})$$ (15)

Where *F*_*in*_ denotes the STFT-derived input feature map and *F*_*out*_ denotes the mixed output. This formulation combines a 3 × 3 depthwise convolution, a 1 × 1 pointwise convolution, batch normalization, and a residual shortcut to preserve local spectral structures. For stride-2 blocks the Token Mixer switches to a downsampling variant with an optional SE attention module that adaptively re-weights channel responses:


$$k=p\left(W_{2}ReLU(W_{1}GAP(F_{in}))\right)$$ (16)

The SE attention module helps the model focus on important frequency channels that contain signal information, improving the model’s ability to detect and classify signals in noisy environments. The Channel Mixer is responsible for feature mixing in the channel dimension, adopting an inverted residual structure, with the mathematical formula as follows:


$$F_{out}=F_{in}+Conv_{1\times 1}\left(GELU\left(Conv_{1\times 1}(expand(F_{in}))\right)\right)$$ (17)

Which includes 1 × 1 point convolution for channel expansion, GELU activation, and channel compression. The Channel Mixer enables the model to learn rich feature representations across different frequency channels, facilitating the discrimination of different modulation types and signal characteristics.

#### Re-parameterization technology.

The re-parameterization technology used by RepViT is the core innovation that enables efficient signal processing. During the training phase, multi-branch structures are used to enhance expressive capability for learning complex signal patterns, and during the inference phase, multi-branches are fused into a single branch through mathematically equivalent transformations: $W_{fused}=\Sigma_{i=1}^{N}\alpha_{i}W_{i}$, where *W*_*i*_ is the convolutional weight of the -th branch, and $\alpha_{i}$ is the fusion weight coefficient. This re-parameterization approach allows the model to achieve high accuracy in signal segmentation while maintaining fast inference speed, which is crucial for real-time signal processing applications.

#### FPN fusion and segmentation head.

**Fig 5:**
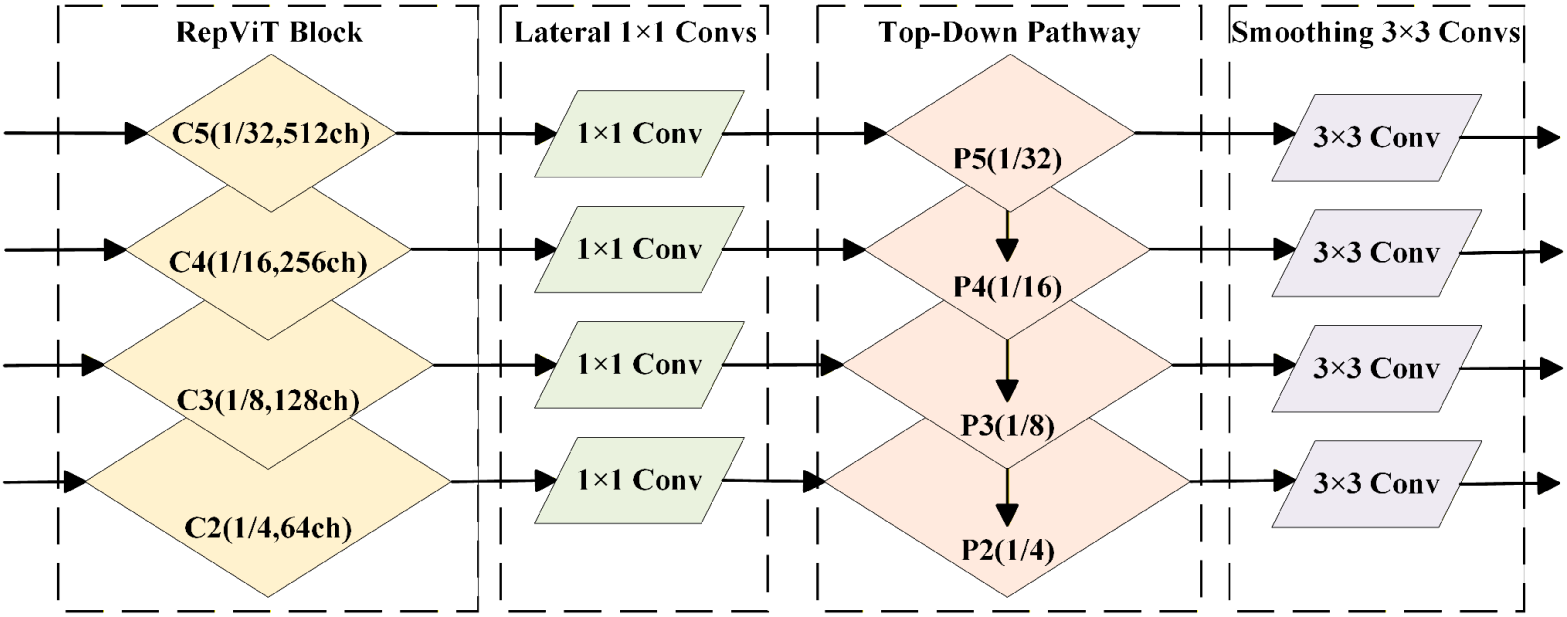
RepViT-based Feature Pyramid Network structure.

The overall network employs a Feature Pyramid Network (FPN) to fuse multi-scale features extracted from spectrogram. The RepViT-based Feature Pyramid Network structure is shown in Fig 5.

As illustrated in Fig 5, features {*F*_1_, *F*_2_, *F*_3_, *F*_4_, *F*_5_} are aligned through lateral 1 × 1 convolutions and fused via the top-down recursion:


$$P_{l}=UpSample(P_{l+1})+Conv(F_{l}^{backbone})$$ (18)

Such that each pyramid level *P*_*l*_ shares the spatial size of *F*_*l*_. The fused maps are upsampled to *H*/4 × *W*/4, concatenated, compressed by 3 × 3/1 × 1 convolutions, and finally upsampled to *H* × *W* to produce the probability map:


$$M=Softmax\left(Conv_{1\times 1}\left(Conv_{3\times 3}\left(Concat\left(UpSample(P_{l})\right)\right)\right)\right)$$ (19)

#### Parameter decoding.

After M is obtained, the parameter decoding stage transforms pixel-level predictions into structured signal parameters through three steps: connected-component analysis, coordinate transformation, and parameter estimation.

**Fig 6:**
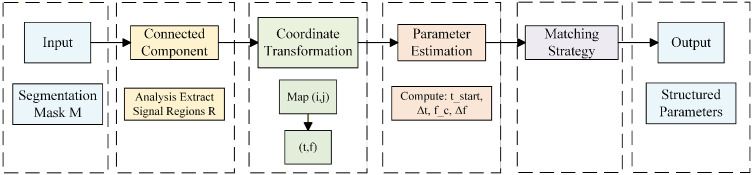
Flowchart for parameter decoding pipeline.

Connected-component analysis extracts distinct signal regions $R=\{R_{1},R_{2},\cdots ,R_{N}\}$ by thresholding the probability map and filtering small noise clusters. Pixel coordinates (*i*,*j*) are then mapped to physical time–frequency coordinates (*t*,*f*) using Eq (3) for frequency mapping and similar transformations for time. For each region *R*_*k*_,*t*he four key parameters are computed: start time and duration from time coordinate extrema, center frequency as the frequency centroid using Eq (3), and bandwidth from frequency coordinate range. Matching with ground truth signals follows Eq (4). This completes the semantic segmentation-to-parameter extraction loop (Fig 6).

For signal semantic segmentation and parameter extraction tasks, RepViT provides multiple model configuration versions: RepViT-M1.1 is suitable for resource-constrained devices requiring real-time signal processing. RepViT-M1.5 balances accuracy and efficiency for practical signal monitoring applications. RepViT-M2.3 pursues higher accuracy for scenarios demanding precise signal parameter estimation. To comprehensively evaluate the performance of RepViT in signal processing tasks, this study selected the following comparison models: RepUNet, ResNet18, and PoolFormer-S12. All comparison models adopt the same FPN and segmentation head design to ensure fair comparison in signal detection, recognition, and parameter extraction tasks.

## 4. Result

Experiments were conducted under eight different noise power levels (−5, 0, 5, 10, 15, 20, 25, 30 dBW), evaluating performance separately for binary and multi-classification tasks. Binary classification distinguishes between signal and background, while multi-classification distinguishes between background and nine different modulation signal types.The binary classification dataset contains 500 samples in total, divided into training, validation, and test sets in an 8:1:1 ratio (400:50:50), resulting in 50 test samples per noise level across 8 noise levels. The multi-classification dataset contains 14,400 samples in total, divided into training, validation, and test sets in an 8:1:1 ratio (11,520:1,440:1,440), resulting in 1,440 test samples per noise level across 8 noise levels. All models were trained with identical configurations and data augmentation strategies to ensure fair comparison. To evaluate the performance of parameter extraction, this paper introduces the Normalized Root Mean Squared Error (NRMSE). For frequency parameters (center frequency, bandwidth), NRMSE is defined as:


$$NRMSE=RMSE/f_{s}$$ (20)

Where *f*_*s*_ is the sampling rate. For time parameters (start time, duration), NRMSE is defined as:


NRMSE=RMSE/T (21)

Where *T* is the complete span of the time window. The evaluation metrics used in this study are summarized in Table 1.

**Table 1:** Evaluation metrics.

Metric Name	Symbol	Description
Intersection over Union	IoU	Intersection over Union ratio for signal categories
Accuracy	Acc	Accuracy for signal categories
Mean Intersection Over Union	mIoU	Average of Intersection over Union for all categories
Mean Accuracy	mAcc	Average accuracy for all categories
Overall Pixel Accuracy	aAcc	Ratio of correctly classified pixels to total pixels
Normalized Root Mean Squared Error	NRMSE	RMSE divided by sampling rate or time window

### Binary classification task results

The binary classification task is divided into signal and background classes. The following table shows the evaluation metrics for all models in the binary classification task. Table 2 provides a comprehensive summary of semantic segmentation performance and parameter extraction errors for the binary classification task.

**Table 2:** Average performance comparison of all models for binary classification task.

Model	Average mIoU(%)	Average mAcc(%)	Average aAcc(%)	Start Time NRMSE	Duration NRMSE	Center Frequency NRMSE	Bandwidth NRMSE
**RepViT-M1.1**	**88.76**	**88.77**	**99.79**	0.003	0.004	**0.081**	**0.016**
RepViT-M1.5	88.66	88.65	99.78	0.003	0.004	0.083	0.016
RepViT-M2.3	87.93	87.93	99.78	0.003	0.004	0.085	0.017
RepUNet	88.68	88.68	99.79	0.003	0.004	0.088	0.017
ResNet18	88.34	88.34	99.78	0.003	0.004	0.087	0.017
PoolFormer-S12	87.57	87.57	99.77	0.003	0.004	0.090	0.017

In the binary classification task, RepViT-M1.1 achieved the best overall performance, although the differences among the six models in terms of average mIoU, average mAcc, and average aAcc are not extremely large, RepViT-M1.1 consistently ranks first, achieving an average mIoU of 88.76%, average mAcc of 88.77%, and average aAcc of 99.79%. This indicates its superior pixel-level segmentation accuracy in signal regions while maintaining near-perfect overall pixel accuracy. RepViT-M1.5 and M2.3 follow closely, also outperforming RepUNet, ResNet18, and PoolFormer-S12 in all three metrics of average mIoU, average mAcc, and average aAcc, demonstrating the feature fusion advantages of the re-parameterized backbone combined with FPN.

In terms of time parameters, the start time NRMSE and duration NRMSE of all models are controlled within the range of 0.3%−0.4%, indicating that all models perform comparably in time-domain boundary characterization with extremely low error levels. In terms of frequency parameters, RepViT-M1.1 achieves a center frequency NRMSE of 8.1% and bandwidth NRMSE of 1.6%, which are 1%−2% lower than ResNet18 and RepUNet, and 10%−11% lower than PoolFormer-S12, indicating that its output mask frequency-domain contours more closely match the true signals, suitable for subsequent connected component matching and parameter recovery. Overall, RepViT outperforms other comparison models in both pixel-level recognition and parameter extraction, demonstrating its comprehensive advantages in signal detection, recognition, and parameter extraction tasks.

**Fig 7:**
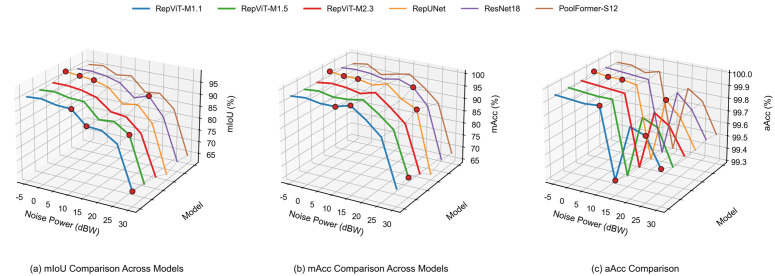
Performance comparison (binary classification): (a) mIoU, (b) mAcc, (c) aAcc.

Fig 7 shows the semantic segmentation performance comparison of all models for binary classification. Subplot (a) shows the average mIoU variation curves of each model under different noise powers. Subplot (b) shows the mAcc variation trend with noise power. Subplot (c) shows the aAcc variation of signal categories under different noise levels.

It is found that under low noise conditions, all models perform excellently, with mIoU exceeding 90%. Under these conditions, RepUNet achieves the highest mIoU of 97.36%, slightly leading, but RepViT-M1.1 follows closely with a small gap. Under medium noise conditions, the performance of all models begins to decline, but RepViT-M1.1 and RepViT-M1.5 can still maintain relatively high performance, with mIoU around 88–89%, outperforming other models (e.g., approximately 1–3 percentage points higher than RepUNet, ResNet18, and PoolFormer-S12). Under high noise conditions, RepViT-M1.1 can still achieve 67.31% mIoU at 30 dBW noise, outperforming RepUNet, ResNet18, and PoolFormer-S12 (e.g., approximately 3–5 percentage points higher), demonstrating its good robustness in extreme noise environments.

Furthermore, RepViT-M1.1’s mIoU curve maintains above 95% under low noise conditions and still maintains approximately 67% under extreme noise conditions, higher than RepUNet, ResNet18, and PoolFormer-S12. The mAcc and aAcc subplots also show an overall leading pattern for the RepViT series, especially under high noise conditions, where RepViT maintains a 3–5 percentage point recognition rate advantage compared to control models. Notably, the aAcc curve shows a consistent slight decline at 15 dBW (approximately 99.33%−99.40%), mainly because the signal energy at this “medium-high noise” level is insufficient to completely suppress noise, causing weak signal pixels to be more easily misclassified as background. Overall, RepViT, through the combination of re-parameterized backbone and FPN, simultaneously maintains higher pixel segmentation accuracy and recognition stability under different noise scenarios.

**Fig 8:**
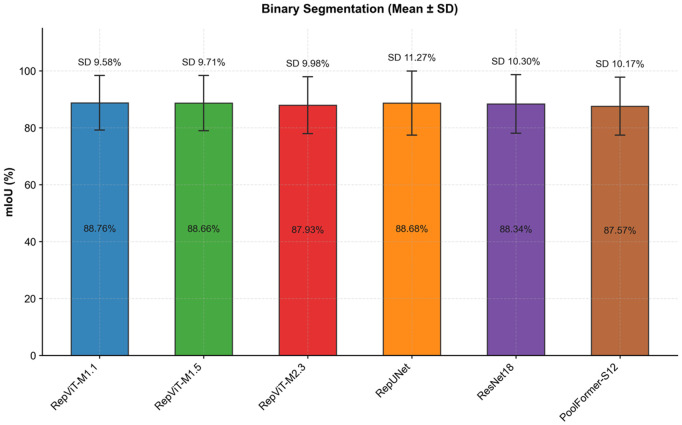
Performance stability comparison (binary classification).

To evaluate the stability of models under different noise environments, this study also calculates the standard deviation of mIoU for each model. A smaller standard deviation indicates stronger adaptability to noise changes and better robustness. Analyzing the stability results from Fig 8, it can be seen that the average standard deviation of the RepViT series is 9.76%, with RepViT-M1.1 having the smallest standard deviation of only 9.58%, indicating minimal performance fluctuation under different noise environments. Among other models, RepUNet has the largest standard deviation of 11.27%, indicating that its performance is more sensitive to noise changes; ResNet18 and PoolFormer-S12 have standard deviations of 10.30% and 10.17%, respectively, also higher than the RepViT series.

This result indicates that RepViT, through re-parameterization technology and efficient architecture design, not only improves accuracy but, more importantly, exhibits excellent robustness, enabling better adaptation to different noise environments.

**Fig 9:**
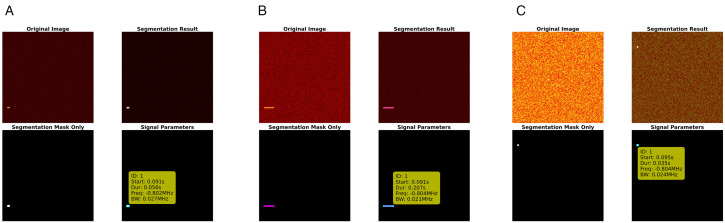
Binary classification results: (a) 0dBW, (b) 15dBW, (c) 30dBW.

To intuitively demonstrate the performance of binary classification models under different noise conditions, Fig 9 selects typical inference results of RepViT under low noise (−5 dBW), medium noise (15 dBW), and high noise (30 dBW) scenarios. Each sample is presented in a four-panel format. Top-left shows the original spectrogram, and top-right shows the overlay of predicted mask and original image, while bottom-left shows the binary segmentation mask, and bottom-right shows automatically extracted values of start time, duration, center frequency, and bandwidth.

Through this example, the complete process of connected component analysis and parameter recovery can be clearly seen. From a visual perception perspective, under low noise conditions, signal contour edges are sharp and background noise is extremely low, and RepViT can completely cover the true signal regions. In the 15 dBW scenario, time-frequency energy begins to diffuse, with local blurring and adhesion, but the model can still maintain mask shapes closely adhering to true boundaries, with only slight expansion at signal endpoints. Under extreme noise at 30 dBW, background energy and signal energy are close, and when observed with the naked eye, some signals are even swallowed by noise, but the model can still capture main energy ridges and maintain coherent detection results, indicating that its segmentation output remains reliable in the main regions. From a system robustness perspective, the parameter panel in the bottom-right corner consistently provides stable start time and center frequency estimates under all three noise levels. Meanwhile, key indicators such as bandwidth and duration do not show severe oscillations under increased noise, confirming the interpretability and engineering usability of the “segmentation first, parameters second” approach in binary classification tasks.

### Multi-classification task results

Compared to binary classification with only background and signals, multi-classification tasks are more challenging, requiring simultaneous distinction of 9 different modulation types (BPSK, QPSK, 8PSK, 16QAM, 64QAM, FM, AM-DSB, AM-SSB, MSK) and background.

Table 3 shows the semantic segmentation performance and parameter extraction accuracy of all models for the multi-classification task. From the perspective of semantic segmentation metrics, RepViT-M2.3 performs best in terms of average mIoU, average mAcc, and average aAcc, outperforming other comparison models. RepViT-M1.1 and RepViT-M1.5 follow closely, with average mIoU of 56.50% and 56.43%, respectively, both outperforming RepUNet, ResNet18, and PoolFormer-S12. In terms of average mAcc, the RepViT series models also perform excellently, with RepViT-M2.3 reaching 65.16%, an improvement of 5.59 percentage points compared to PoolFormer-S12. In terms of overall pixel accuracy (aAcc), all models achieve above 99.86%, indicating that the models have high accuracy in overall classification of both background and signals.

**Table 3:** Average performance comparison of all models for binary classification task.

Model	Average mIoU(%)	Average mAcc(%)	Average aAcc(%)	Start Time NRMSE	Duration NRMSE	Center Frequency NRMSE	Bandwidth NRMSE
**RepViT-M2.3**	**56.91**	**65.16**	**99.88**	0.018	**0.010**	0.087	0.006
RepViT-M1.1	56.50	64.40	99.88	0.028	0.014	0.091	0.005
RepViT-M1.5	56.43	64.59	99.88	0.006	0.010	0.093	0.005
ResNet18	52.30	61.40	99.87	0.007	0.010	0.102	0.005
RepUNet	51.88	61.23	99.88	0.012	0.016	0.155	0.007
PoolFormer-S12	50.11	59.57	99.86	0.012	0.015	0.086	0.008

From the perspective of parameter extraction metrics, RepViT-M1.5 achieves the lowest value of 0.6% in start time NRMSE, outperforming other models; in duration NRMSE, RepViT-M2.3, RepViT-M1.5, and ResNet18 all achieve the lowest value of 1%. In center frequency NRMSE, PoolFormer-S12 achieves the lowest value of 8.6%, while RepViT-M2.3 is 8.7%, which is 43.9% lower than RepUNet and 14.7% lower than ResNet18. In bandwidth NRMSE, ResNet18, RepViT-M1.1, and RepViT-M1.5 all achieve the lowest value of 0.5%, while RepViT-M2.3 is 0.6%, which is 25.0% lower than PoolFormer-S12, demonstrating the comprehensive advantages of the RepViT series in signal detection, recognition, and parameter extraction under multi-modulation type scenarios.

**Fig 10:**
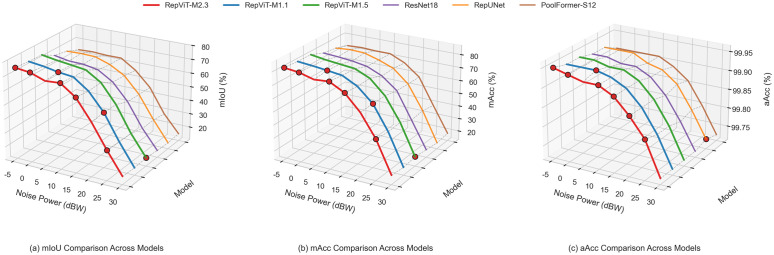
Performance comparison (multi-classification): (a) mIoU, (b) mAcc, (c) aAcc.

Fig 10 shows the mIoU variation trends of all models in the multi-classification task under different noise power levels. It can be seen that under low noise conditions, the RepViT series models perform excellently, with mIoU exceeding 70%. RepViT-M2.3 achieves the highest mIoU of 75.69% under −5 dBW noise conditions, outperforming RepUNet, ResNet18, and PoolFormer-S12. Under medium noise conditions, the performance of all models begins to decline, but the RepViT series can still maintain relatively high performance. Under 15 dBW conditions, RepViT-M2.3 achieves 64.04% mIoU, outperforming other models. As for high noise environments, this is the most challenging scenario for multi-classification tasks. RepViT-M2.3 can still achieve 32.34% mIoU under 25 dBW noise, outperforming RepUNet, ResNet18, and PoolFormer-S12. Under extreme noise conditions (30 dBW), the performance of all models declines, but RepViT can still maintain relative advantages.

**Fig 11:**
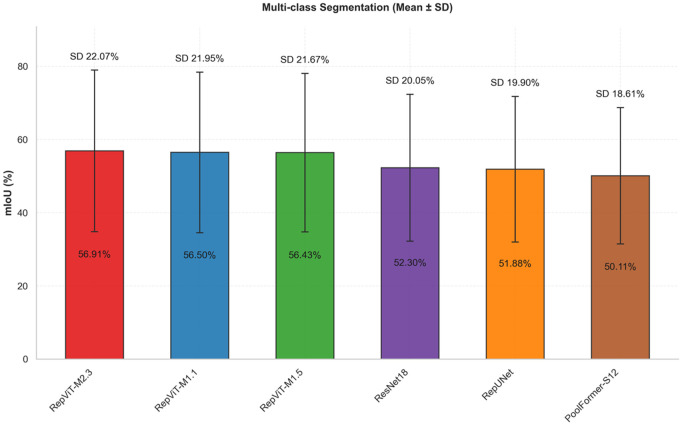
Performance stability comparison (multi-classification).

To evaluate the stability of models in the multi-classification task, this study also calculates the standard deviation of mIoU for each model in multi-classification. From the stability analysis results, as shown in Fig 11, for the RepViT series in multi-classification, the standard deviation ranges from 21.67% to 22.07%, with RepViT-M1.5 having the smallest standard deviation, indicating relatively smaller performance fluctuations under different noise environments. For other models in multi-classification, RepUNet has the largest standard deviation, while ResNet18 and PoolFormer-S12 have standard deviations of 20.05% and 18.61%, respectively. Although RepViT’s standard deviation is slightly higher than other models, this is because it can still maintain high performance under high noise conditions while outperforming other models under low noise conditions, resulting in a larger performance range.

**Fig 12:**
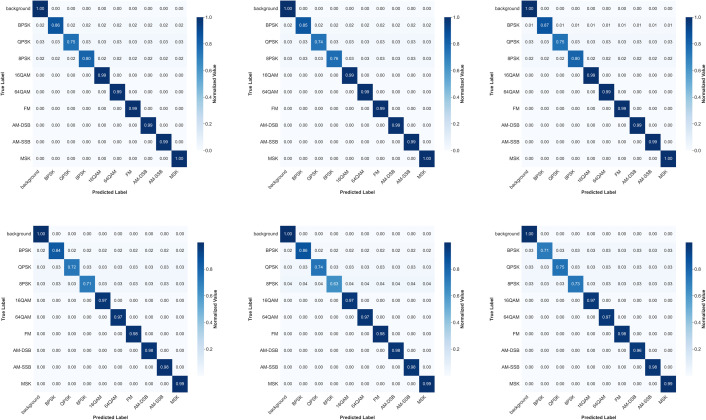
Confusion matrices of all models at 0dBW noise power.

To deeply analyze the recognition capabilities of different models for the 9 modulation types, confusion matrices for each model are generated under different noise power levels in the study, as shown in Fig 12 (using 0dBW noise power as an example), which can intuitively demonstrate the classification error patterns between different categories.

From the confusion matrix of the RepViT-M1.1 model at 0dBW noise power, it can be observed that under high signal-to-noise ratio conditions, all models achieve relatively high recognition accuracy for the 9 modulation types, with the RepViT series models generally achieving higher IoU across all categories than other models. Confusion mainly occurs between signals with similar modulation types, such as BPSK and QPSK, 8PSK and 16QAM, etc. The recognition accuracy of digital modulation types is generally higher than that of analog modulation types. Under medium noise conditions, the classification accuracy of models begins to decline, but RepViT can still maintain relatively high performance. The degree of confusion increases, particularly between digital modulation types, such as 16QAM and 64QAM, and between analog modulation types, such as AM-DSB and AM-SSB. Under low signal-to-noise ratio conditions, the classification accuracy of models declines, but RepViT can still maintain relatively high performance, especially in signal detection. Under extreme noise conditions, most models tend to misclassify signals as background, but RepViT’s misclassification rate is relatively low.

Under 0dBW noise power conditions, the confusion matrices of the six models exhibit the following commonalities and differences. All models can distinguish the nine modulation signal types relatively well under high signal-to-noise ratios, with confusion mainly occurring between categories with similar modulation forms. The three RepViT models of different scales all achieve higher diagonal IoU values across all categories than other models, especially RepViT-M2.3, which shows the most outstanding distinction for high-order QAM and MSK, maintaining relatively clear boundaries even between similar modulation types.

RepUNet’s overall diagonal values are slightly lower than RepViT, and confusion between 16QAM and 64QAM, and between AM-DSB and AM-SSB is more pronounced. The recall rate for analog modulations shows the largest decline. ResNet18 is similar to RepUNet, with misclassifications between digital modulations mainly concentrated in high-order QAM, low recognition rates for analog modulations, and a slightly higher background misclassification rate than RepViT. As for PoolFormer-S12, the diagonal values for categories such as AM-SSB and MSK further decrease, indicating insufficient capability in scenarios requiring fine spectral features. Digital modulation recognition is basically maintained, but there is more confusion between categories.

Overall, under the baseline noise environment of 0dBW, the RepViT series shows clear advantages in metrics such as IoU and recall rate for the nine signal types. Other models show higher confusion between similar modulation types, with particularly weak recognition of analog modulations. Under different signal-to-noise ratios, the recognition difficulty of specific modulation types is high.

The reason for these results is that the recognition difficulty of specific modulation types is high. For example, AM-DSB and AM-SSB, due to their similar spectral characteristics, exhibit certain confusion in all models, especially under low noise conditions. Digital modulation types achieve high recognition accuracy under low noise conditions, but accuracy declines under high noise conditions. Analog modulation types have relatively high recognition difficulty under all noise conditions, especially under high noise conditions.

**Fig 13:**
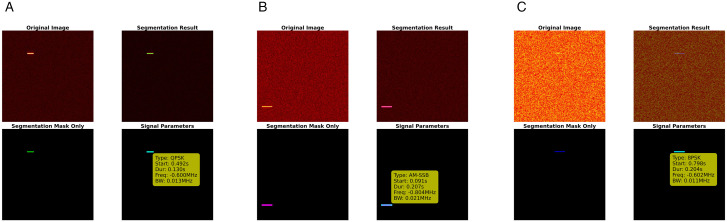
Multi-classification results: (a) 0dBW, (b) 15dBW, (c) 30dBW).

This study also extracts signal parameters from RepViT multi-classification segmentation results, including start time, duration, center frequency, and bandwidth. Through connected component analysis and frequency calibration, automatic parameter extraction is successfully achieved, as shown in Fig 13. Fig 13 shows the multi-classification segmentation results and parameter extraction effects of the RepViT model under three typical noise environments: low noise (-5dBW), medium noise (15dBW), and high noise (30dBW).

From a visual perception perspective, under low noise conditions, signal features in the spectrogram are clearly visible, and RepViT can accurately identify and segment signal regions of different modulation types, with segmentation masks highly matching true signal boundaries, and time-frequency features of various modulation signals can be accurately captured. Under medium noise conditions, signal features in the spectrogram begin to blur, with slight noise interference appearing at some signal edges, but RepViT can still maintain high segmentation accuracy, with most signal regions still correctly identified and localized, with only slight boundary deviations in small overlapping areas. Under high noise conditions, signal features in the spectrogram are severely degraded, background noise is enhanced, and the contrast between signals and noise is greatly reduced. At this point, although RepViT shows slight mis-segmentation at some detailed boundaries, it can still accurately detect main signal regions overall, demonstrating good visual consistency.

From a system robustness perspective, RepViT exhibits excellent performance stability under different noise levels. Under low noise conditions, the system can fully utilize clear signal features to achieve near-perfect segmentation accuracy, providing a high-quality foundation for subsequent parameter extraction. Under medium noise conditions, although signal features are degraded, high segmentation accuracy can still be maintained, and parameter extraction accuracy, while slightly decreased, remains within acceptable ranges, demonstrating the system’s good adaptability to moderate noise interference. Under extreme high noise conditions, although segmentation accuracy decreases, RepViT can still maintain basic signal detection capability, with key signal regions (such as the main body of signals) still accurately identified, avoiding complete failure, demonstrating the system’s survival capability and robustness in extreme environments. This stable performance across noise levels indicates that RepViT’s re-parameterized architecture and Feature Pyramid Network design can effectively cope with noise interference of different intensities, providing reliable technical support for complex electromagnetic environments in practical applications.

**Fig 14:**
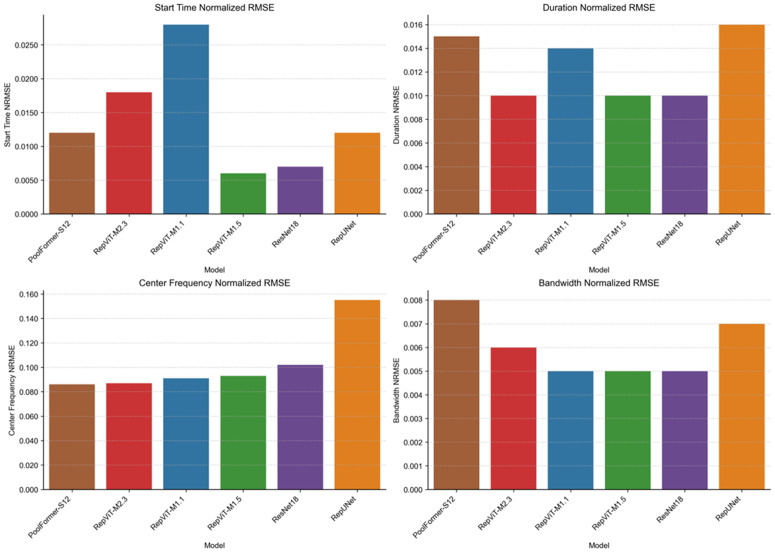
Parameter extraction NRMSE comparison (multi-classification).

To further quantify the advantages of each model in parameter extraction tasks, this paper conducts a centralized visualization analysis of time and frequency parameter errors across multiple models. Fig 14 shows the NRMSE comparison of all models in four key parameter extraction tasks. The four subplots correspond to the NRMSE values of start time, duration, center frequency, and bandwidth, respectively. All errors are normalized according to their respective ranges to eliminate dimensional effects and facilitate cross-parameter comparison.

From the overall trend, the RepViT series models demonstrate competitive performance across all four parameters. For time-domain parameters, all models achieve relatively low NRMSE with small inter-model differences, while RepViT shows more prominent advantages in frequency-domain parameters. Among them, PoolFormer-S12 performs optimally in center frequency extraction, with NRMSE reaching 8.6%, while RepViT-M2.3 is 8.7%, which is 43.9% lower than RepUNet and 14.7% lower than ResNet18. In bandwidth extraction, RepViT-M1.5 and RepViT-M2.3 achieve NRMSE of 0.5% and 0.6%, respectively, which are 37.5% and 25.0% lower than PoolFormer-S12, respectively. Notably, all models control start time NRMSE within the range of 0.6% to 2.8%, and duration NRMSE within the range of 1% to 1.6%, indicating high time-domain boundary characterization accuracy with small differences between models. In frequency-domain parameters, RepViT’s advantages are more pronounced, with center frequency errors approximately 14%−44% lower than comparison models, validating the effectiveness of its re-parameterized architecture in spectral feature extraction.

**Fig 15:**
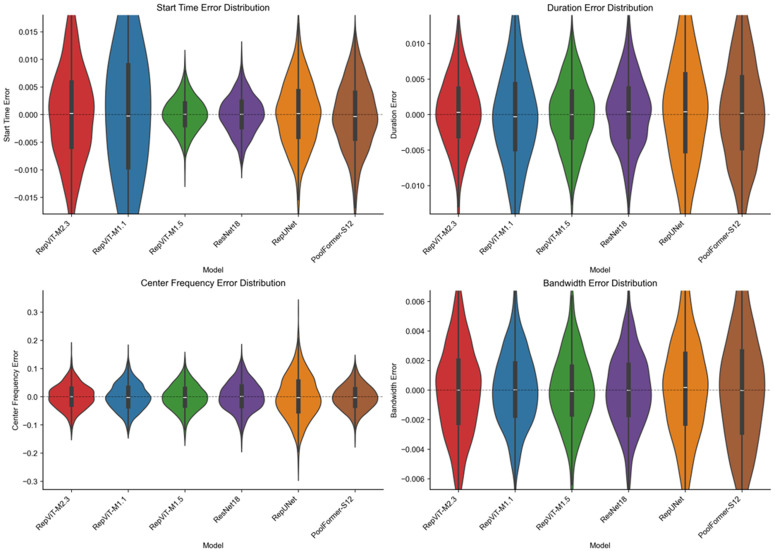
Error distribution violin plots (multi-classification).

Fig 15 shows the error distribution characteristics of each model across four parameters through violin plots, intuitively reflecting the concentration and dispersion of prediction errors. From the distribution morphology, the RepViT series models show more concentrated error distributions closer to the zero-error line, indicating higher stability and consistency in their prediction results. In contrast, RepUNet and ResNet18 exhibit obvious long-tail characteristics in their error distributions, with more outlier samples at extreme error values. PoolFormer-S12 has the most dispersed error distribution, particularly showing large variance in bandwidth estimation tasks. For time-domain parameters, all models show good symmetry and concentration in their error distributions, further confirming the robustness of time-domain boundary detection. For frequency-domain parameters, RepViT’s error distribution is narrower, with error standard deviations for center frequency and bandwidth approximately 20% and 25% lower than PoolFormer-S12, respectively, demonstrating its parameter estimation advantages in complex spectral environments.

**Fig 16:**
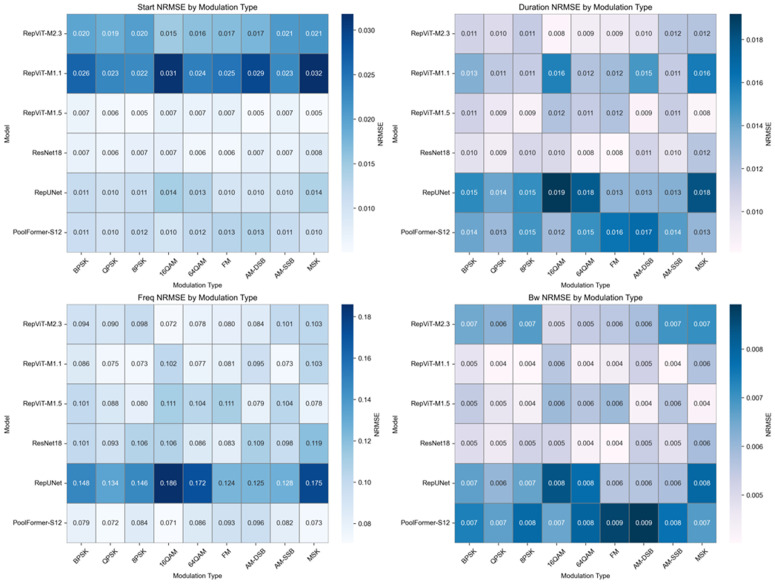
Heatmap comparison of parameter extraction NRMSE.

Fig 16 shows the parameter extraction NRMSE performance of different models across nine modulation types in heatmap form, revealing the adaptive differences of models to different modulation signals. From the color gradient of the heatmap, it can be seen that the RepViT series models maintain light-colored regions (low NRMSE) for most modulation types, particularly excelling in digital modulation types. For time-domain parameters (start time and duration), all models demonstrate relatively low NRMSE with small inter-model differences, indicating high accuracy in temporal boundary characterization. For frequency-domain parameters (center frequency and bandwidth), the RepViT series models show more prominent advantages, maintaining consistently lower NRMSE across most modulation types compared to other models.

In contrast, analog modulation types, due to relatively complex spectral features and unclear boundaries, show increased NRMSE for all models, but RepViT still maintains relative advantages. Notably, RepViT-M2.3 shows particularly outstanding advantages in center frequency extraction, with reductions of approximately 43.9% and 14.7% compared to RepUNet and ResNet18, respectively. The bandwidth NRMSE advantages for complex modulation types such as high-order QAM and MSK are also notable, with reductions of approximately 25%−38% compared to PoolFormer-S12, indicating its ability to better handle spectrally overlapping and morphologically complex signal scenarios.

Overall, RepViT demonstrates more balanced performance on both digital and analog modulation signals, providing reliable technical support for parameter extraction in multi-modulation type scenarios, proving the comprehensive performance advantages of RepViT in multi-signal semantic segmentation and parameter extraction.

To evaluate the computational efficiency of the proposed frame, we report the model size (number of parameters), computational complexity (FLOPs), and inference time for each model variant. Table 4 summarizes the complexity metrics and inference performance on spectrograms of size 1024 × 741 pixels.

**Table 4:** Model complexity and inference time.

Model	Parameters (M)	FLOPs (G)	GPU Time (ms, RTX 4090)	CPU Time (ms, Intel i7)
RepViT-M1.1	12.0^*a*^	82.2^*b*^	11.1	643.9
RepViT-M1.5	17.8^*a*^	96.6^*b*^	18.6	983.7
RepViT-M2.3	26.7^*a*^	131.3^*b*^	21.7	1430.6
RepUNet	0.2^*e*^	6.1^*b*^	6.9	560.7
ResNet18	16.6^*d*^	122.5^*b*^	9.0	536.2
PoolFormer-S12	12.0^*c*^	38.0^*b*^	4.2	334.0

^a^RepViT series: RepViT backbone + FPN segmentation head.

^b^FLOPs are calculated based on input size 1024 × 741.

^c^PoolFormer-S12: PoolFormer-S12 backbone + PoolFormerHead segmentation head.

^d^ResNet18: ResNet18 backbone + ResNet18HeadModule segmentation head.

^e^RepUNet: RepUNet backbone + RepUNetHead segmentation head.

Inference times are measured on RTX 4090 GPU and Intel i7 CPU with batch size 1 (100 runs, 10 warmup runs).

As shown in Table 4, RepViT variants demonstrate efficient performance on RTX 4090 GPU. RepViT-M1.1 achieves an inference time of 11.1 ms with 12.0M parameters and 82.2 GFLOPs, RepViT-M1.5 achieves 18.6 ms with 17.8M parameters and 96.6 GFLOPs, and RepViT-M2.3 achieves 21.7 ms with 26.7M parameters and 131.3 GFLOPs for input size 1024 × 741. All RepViT variants employ the FPN (Feature Pyramid Network) segmentation head, which consists of an FPN neck for multi-scale feature fusion and an FPNHead for final prediction. The FPN head effectively combines features from different scales through lateral connections and progressive upsampling, contributing approximately 3.8−3.9M parameters to the total model size. RepUNet achieves the lowest parameter count of 0.2M with 6.9 ms GPU inference time and 6.1 GFLOPs, demonstrating exceptional parameter efficiency. RepUNet uses an end-to-end architecture with a lightweight RepUNetHead decoder that employs progressive upsampling and skip connections. ResNet18 achieves 9.0 ms GPU inference time with 16.6M parameters and 122.5 GFLOPs, demonstrating competitive speed while maintaining higher computational complexity. ResNet18 employs a custom ResNet18HeadModule segmentation head with multi-scale feature fusion. PoolFormer-S12 achieves the fastest GPU inference time of 4.2 ms with 12.0M parameters and 38.0 GFLOPs, demonstrating its efficiency advantage in terms of raw speed. PoolFormer-S12 uses a simplified FPN-style head (PoolFormerHead) with lateral connections and progressive upsampling. However, RepViT-M1.1 offers a better balance between speed and accuracy, achieving competitive segmentation performance while maintaining reasonable inference time. All models demonstrate their suitability for real-time signal processing applications. The complete segmentation models (backbone + segmentation head) maintain competitive efficiency while providing pixel-level segmentation capabilities.

By synthesizing the experimental results of binary classification, multi-classification, and parameter extraction, the following main conclusions can be drawn. In semantic segmentation tasks, the RepViT series models demonstrate outstanding performance in both binary and multi-classification scenarios. For binary classification tasks, RepViT-M1.1 achieves 88.76% average mIoU, performing best among all models, and demonstrates excellent stability and robustness with a standard deviation of 9.58%. It still maintains 67.31% mIoU under extreme noise conditions, outperforming comparison models (e.g., 3–5 percentage points higher than RepUNet, ResNet18, and PoolFormer-S12 under 30 dBW noise). For multi-classification tasks, RepViT-M2.3 achieves 56.91% average mIoU, improving by 4.61, 4.61, and 6.80 percentage points compared to RepUNet, ResNet18, and PoolFormer-S12, respectively. Even under 25 dBW noise conditions, it can achieve 32.34% mIoU, and maintains 16.61% mIoU under extreme 30 dBW noise, both higher than other models, demonstrating the ability to finely distinguish multiple modulation types. In low noise scenarios, RepViT-M2.3 achieves 75.69% mIoU, outperforming comparison models (e.g., approximately 4–6 percentage points higher than RepUNet, ResNet18, and PoolFormer-S12).

In parameter extraction tasks, the RepViT series demonstrates advantages in normalized root mean squared error (NRMSE), with more prominent improvements observed in frequency-domain parameters (center frequency and bandwidth) compared to time-domain parameters (start time and duration).

From the perspective of time-domain parameters, all models control start time NRMSE within the range of 0.6%−2.8%, and duration NRMSE within the range of 1%−1.6%, indicating high time-domain boundary characterization accuracy with small differences between models. Among them, RepViT-M1.5 achieves the lowest value of 0.6% in start time NRMSE, and RepViT-M2.3, RepViT-M1.5, and ResNet18 all achieve the lowest value of 1% in duration NRMSE.

From the perspective of frequency-domain parameters, RepViT’s advantages are more prominent: in binary classification tasks, RepViT-M1.1 achieves center frequency NRMSE of 8.1% and bandwidth NRMSE of 1.6%, which are 10.0% and 5.9% lower than PoolFormer-S12, respectively. PoolFormer-S12, all outperforming RepUNet, ResNet18 and PoolFormer-S12 (with center frequency NRMSE reductions of 43.9% and 14.7% compared to RepUNet and ResNet18, respectively, and bandwidth NRMSE reduction of 25.0% compared to PoolFormer-S12). The error distribution analysis shows that the RepViT series models have more concentrated error distributions closer to the zero-error line, with error standard deviations for center frequency and bandwidth approximately 20% and 25% lower than PoolFormer-S12, respectively, demonstrating their parameter estimation advantages in complex spectral environments. Fine-grained comparisons across different modulation types show that RepViT performs excellently on digital modulation types. On analog modulation types, although all models show increased NRMSE, RepViT still maintains relative advantages, with particularly outstanding bandwidth NRMSE advantages for complex modulation types such as high-order QAM and MSK, with reductions of approximately 25%−38% compared to PoolFormer-S12. Combined with the multiple size configurations provided by the models, users can flexibly choose according to different resource and accuracy requirements.

Overall, RepViT demonstrates high accuracy, stability, and robustness in multi-signal semantic segmentation and parameter extraction tasks, achieving competitive performance in both time-domain and frequency-domain parameter extraction, providing solid technical support for intelligent analysis of wideband signals in complex electromagnetic environments.

## 5. Conclusion

This paper presents a unified end-to-end frame for wideband signal analysis, centered on semantic segmentation-driven recognition and parameter extraction. The proposed approach transforms wideband signals into time-frequency representations, encodes them as color-mapped spectrograms, and processes them with a RepViT-FPN network for pixel-level segmentation. The key signal parameters—including start time, duration, center frequency, and bandwidth—are then recovered through connected-component analysis and frequency calibration. Extensive experiments on a dataset comprising nine modulation types across eight noise levels demonstrate that the frame achieves robust signal recognition and accurate parameter extraction. Comparative evaluations with similar network architectures confirm that the proposed method maintains stable performance under varying noise conditions, demonstrating improved robustness. In summary, this work effectively enhances the performance and automation level of wideband signal processing. The proposed frame provides a valuable technical reference for the design of future intelligent spectrum sensing and monitoring systems. It should be noted that the current evaluation is limited to synthetic data with additive white Gaussian noise (AWGN) and nine modulation types. Future work will extend the evaluation to more realistic interference scenarios, such as single-tone jamming, modulated interference signals, and co-channel interference, and investigate domain adaptation techniques for real-world signals with channel fading and hardware impairments.
